# Relation between perceived emotional intelligence and social factors in the educational context of Brazilian adolescents

**DOI:** 10.1186/s41155-019-0139-y

**Published:** 2020-01-08

**Authors:** M. Vaquero-Diego, P. Torrijos-Fincias, M. J. Rodriguez-Conde

**Affiliations:** Department of Didactics, Organization and Research Methods, Institute of Education Sciences, University of Salamanca, Paseo Canalejas, 169, 37008 Salamanca, Spain

**Keywords:** Emotional intelligence, Adolescence, Gender, TMMS-24

## Abstract

**Background:**

The study of emotional intelligence (EI), demographics, and family factors of adolescent high school students allows us to appraise adolescents’ skills for their academic and vocational training. The objectives of the study focus on whether there is any relationship between context variables such as gender, age of parents, or work activity, and self-perception of emotional intelligence.

**Methodology:**

This study sampled 11.370 participants, aged between 12 and 17 years, in the 7th and 9th years of fundamental education, and the 3rd year of upper secondary education. Data from this study comes from students enrolled in the SESI schools of the City of Sao Paulo. To examine the data, we applied the TMMS-24 test to statistical analysis where gender relates to the three dimensions of perceived emotional intelligence (PEI): attention, clarity, and emotional repair.

**Results:**

The results obtained allow us to show how teenagers are perceived with respect to three dimensions: attention, clarity and emotional repair, and thereby extrapolating the need to continue the promotion of emotional education in schools.

**Conclusions:**

Our findings suggest that the application of the Brazilian version of the TMMS-24 in training programs in PEI must consider a whole series of sociocultural aspects. These aspects should start with a series of initial measures that allow for the perceptions of participants to be observed, and to extend onward to influence the willingness of the beneficiaries to participate in this type of intervention. Provided the intervention is anchored in a solid theoretical base, and executed under a rigorous study, its efficacy can be verified.

## Background

The concept of emotional intelligence (EI) refers to the capability of individuals to recognize their own emotions and the mental ability to understand human relations as a key element in the educative process (Campo et al., 2015). Research related to this field shows that the EI influences behavior and self-perception of well-being. It has a fundamental role in social interactions, the contents of thought, and the processes involved in forming them (Brackett et al., 2011; Zurita-Ortega et al., 2018). Therefore, the concept of EI has implications on indicators of adjustment and well-being relating to one’s health and the ability to cope with life’s vital challenges (Vergara et al., 2015). EI is recognized by the scientific community from the contributions of renowned researchers such as Salovey and Mayer, 1990.

**Fig 1:**
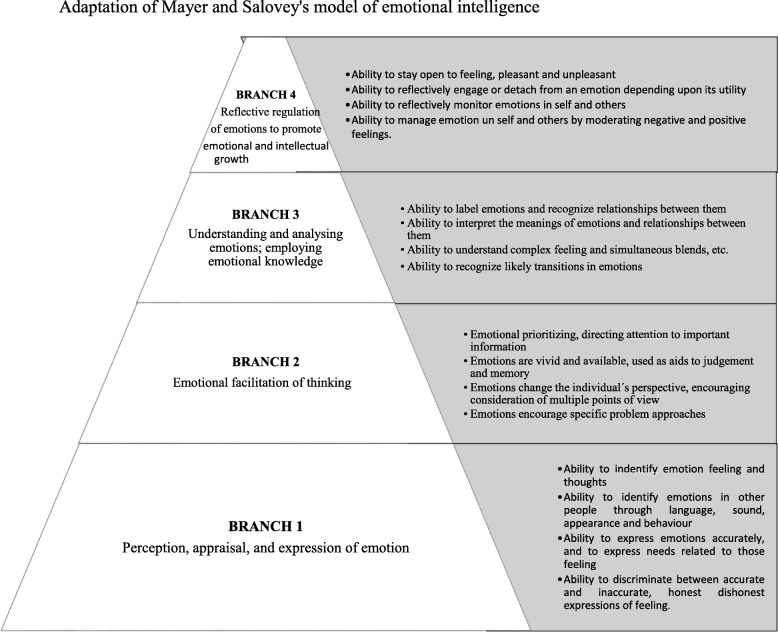
Adaptation of the IE Model by Mayer (1997)

There are two theoretical models of EI can be distinguished based on current scientific literature and its theoretical nature from which it starts. The first, models based on the processing of emotional information, facilitating the use of our own emotions for the management of a more intelligent thinking and its more effective reasoning; from which EI is conceived as the ability to perceive, understand, manage, and regulate emotions, both their own and those of others such as with Mayer (1997) (Fig. 1), and second, the so-called mixed based on personality traits such as the Bar-On models (2000) that describe a cross-section of interrelated socio-emotional competencies, as well as the skills and facilitators that would affect intelligent behavior (Fernández-Berrocal and Pacheco, 2005; Goleman, 1996; Pena Garrido & Repetto Talavera, 2017).

These authors statement that EI was conceived as a set of skills hypothesized to contribute to the accurate appraisal and expression of emotion in oneself and others, the effective regulation of emotion in self and others and the use of feelings to motivate, plan and achieve in one's life (Salovey and Mayer, 1990). This model of Mayer and Salovey contemplates four capacities: one, perception and expression of emotions; two, emotional facilitation of thought; three, understanding and analyzing emotional information; and four, the regulation of emotions.

From this theory, numerous definitions have been proposed for the term EI; however, the meeting point between the different theories and the acceptance of the series of emotional competencies must be well understood and that constituted a crucial factor to favor the adaptation and development of the person in all areas and vital cycles, which can be developed (Durlak et al., 2011).

### The implication of EI in education

The role given to the area of emotional intelligence (EI) in the social sciences as a key aspect and guarantee for well-being, contemplating a series of capacities that are related to academic performance or the improvement of coexistence in the classroom and without a doubt, they are a judge for the success both in the academic and professional fields (Bar-On & Parker, 2000; Dolev & Leshem, 2017; Gilar-Corbi et al., 2018; Gutiérrez-Moret et al., 2016). Scientific studies have established the fact that high levels in these capacities have a causal relationship to better physical and mental health, greater well-being, as well as better social functioning and school performance (Fernández-Berrocal and Ruiz Aranda, 2017). Educational centers, and more specifically schools, have focused on the development of the academic aspects of education, while often neglecting the social and emotional aspects that impact the proper functioning of the classroom, not to mention students’ personal and social well-being (Ghanizadeh & Royaei, 2015; Madalinska-Michalak, 2015).

### The observation of the EI in adolescents

Adolescence is considered a crucial period in a person’s emotional growth. It is when the need to develop emotional competencies and strengths for well-being manifest themselves, and the response patterns to different situational triggers are apparent (Arias & Zafra, 2011).

There are studies that report that adolescents have a high rate, approximately 10 to 20% of the general population, in the prevalence of problems related to emotional disturbances typical of anxiety and depression disorders (Salavera et al., 2019).

Leaders in education who interact with students recognize that the family plays a very important role in their students’ lives. There is a risk to the student when the structure and the dynamics of the family are factors that lead to a crisis or socio-emotional problems. Likewise, schools and youth educational centers can help with socio-emotional difficulties through keeping and enhancing healthy relations with other classmates and teachers. Consequently, educational centers have a key role in the development and the self-affirmation of a student’s personality and self-image and are a central aspect in one’s evolution as a human being (López de Dicastillo Rupérez, N., Iriarte Redín, C. y González Torres, M., 2006).

From the standpoint of the multitude of changes and the need for biopsychosocial adaptations through which the adolescent might passes, they respond to moments where it is demanded and tend to violate norms and have problems to maintain the order demanded**. (**Fernández-Berrocal & Extremera, 2006; Mayer, 1997).

A considerable amount of research interest has focused on the relationship between variables such as emotional competence and self-esteem, psychological well-being, mental health, and life satisfaction (Furr & Funder, 1998; Huebner, 1991; Montes-Berges & Augusto-Landa, 2014). Yet, over the past few years, we have seen that there is a lack of studies showing evidence of adolescents’ self-perceived needs according to their socio-emotional development or Perceived emotional intelligence (PEI) (Toussaint et al., 2015). For this reason, it is necessary to carry out experimental studies in this field in order to see to what degree they support the theoretical models, which are not well understood.

There are currently psychometric instruments based on the Meta-Mood Scale (TMMS-24) models that can be applied as a test (Fernandez-Berrocal et al., 2004) allowing the assessment of emotional intelligence. The TMMS-24 is a useful instrument in evaluating an adolescent’s PEI (Parker et al., 2004). The lack of data for the application of PEI assessments using these instruments and methods in developing countries leads study groups in the social sciences to use these tools in young students in high school (Saucedo et al., 2018). Besides acting as a model for translation and adaptation of the instrument to their educational systems, obtaining data from the application of these instruments may contribute to a better understanding of PEI in adolescents.

In that scenario, the present study must show the main objectives to evaluate the PEI of adolescents in network high schools of São Paulo City in Brazil. This school network is under the direction of the Industrial Social Service (SESI-SP) that is a state organization of private law and structured on a federative basis to provide social and educational assistance. The institution allowed us access and data collection in schools.

Based on the premise of the influence from some variables of contextual factors such as gender, course level, age and parental work activity, we propose the following two initial hypotheses: (1) there are significant differences in adolescents’ self-perceived abilities related to gender, age, and parents’ professional activities, and (2) the variable such as gender, age, and parents’ professional activities influence in the development of emotional competences in adolescents at network high school (SESI-SP).

## Methods

### Participants

The total of sample in the study was established from the entirety of the students at network high school (SESI-SP), based on the available subjects enrolled in the courses in the 7th and 9th year of fundamental education, and the 3rd year of secondary education; the criterion to include these courses is because they are groups evaluated through the School Performance Evaluation System of the State of São Paulo (SARESP), with the anticipation of conducting possible research in the future and because the educational courses correspond to the 3 stages of adolescence considered by authors such as Aberastury (2002) and Blos (1986). Of a total of 14,000 students to whom the questionnaire was sent, the response was 81%, of whom 5,584 were male students (49.1%) and 5,699 were female (50.1%), presenting a very balanced proportion by gender. There was a total of 11370 responses, of which complete data represents *n* = 11283. This study meets the ethical considerations of the Declaration of Helsinki and the criteria of ethical research applied to human beings, and the director of the Education Division at SESI-SP authorized the conducting of this investigation, as well as the gathering of parental authorizations of test subjects.

### Design and data analysis

The proposed research design is of a quantitative nature, developed through a descriptive-correlational study that will allow us to know the distribution of the variables, as well as the inter-correlation between them (Alzina, 2004; Arnal et al., 1992). The study of the data collected after the application of the TMMS-24 test was carried out through statistical analyzes supported by the statistical program SPSS version 23 (Campus License of the University of Salamanca). Based on the first descriptive analysis and characteristics of the variables, we proceeded to study the relationship between them, using techniques such as chi-square statistics, level of significance, and effect size because they are discrete variables.

### Variables

The variables that constitute this study collected through the online questionnaire are the 24 corresponding TMMS-24 items, variable for gender, course levels, and parental employment status.

According to age, 20.6% of 7th grade students participated in basic education (average age 11.96 years), 38.0% of 9th grade students (average age 13.60 years), and 41% of students in the third year of secondary education (average age of 16.45 years).

The sociodemographic characteristics of the sample, as evidenced in Table 1, show similar percentages in terms of gender and occupation, reporting that 72% of mothers work compared to 88% of fathers. However, there are slightly higher percentages of mothers who are in a situation of professional inactivity (Table 2), i.e., mainly engaged in household chores.

**Table 1:** Interpretation of the scores of TMMS-24 of Fernandez-Berrocal et al., 2004

	Men	Women	Valuation
Attention = sum of variables from 1 to 8	≤ 21	≤ 24	Low. should improve
	From 22 to 32	From 25 to 35	Suitable, medium
	≥ 33	≥ 36	High, should improve
Clarity = sum of variables from 9 to 16	≤ 25	≤ 23	Low, should improve
	From 26 to 35	From 24 to 34	Suitable, medium
	≥ 36	≥ 35	Excellent, high
Repair = sum of variables from 16 to 24	≤ 23	≤ 23	Low, should improve
	From 24 to 35	From 24 to 34	Suitable, medium
	≥ 35	≥ 35	Excellent, high

Low, should improve (L); suitable, medium (S); high (H)

**Table 2:** Sample description, gender, level, and employment situation of parent data

		*f*	%
Gender	Men	5584	49.1
	Women	5699	50.1
	Sample error	87	0.8
Level of study	7th fundamental year (12–13 years old)	2339	20.6
	9th fundamental year (14–15 years old)	4323	38.0
	3rd middle school (17–18 years old)	4708	41.4
	Sample error	0	0
Situation (father)	Work	10054	88.4
	DK/NR	71	0.6
	No work	1158	10.2
	Sample error	87	0.8
Situation (mother)	Work	8230	72.4
	DK/NR	46	0.4
	No work	3007	26.4
	Sample error	87	0.8

*DK* don’t know, *NR* no reply

### Instrument

For this investigation, the Spanish Modified Version of the Trait Meta-Mood Scale: TMMS-24 was used (Fernandez-Berrocal et al., 2004). This is a reduced version of the Trait Meta-Mood Scale: TMMS 48 of the research group of Salovey et al. (1995). It is composed of the 3 dimensions of the original scale: (1) attention to emotions, perception skills, and the ability to detect and express one's feelings properly; (2) emotional clarity, related to the understanding of emotions; and (3) emotional repair, meaning the ability to regulate emotional states. The reliability for each component obtained in this investigation by Mayer and Salovey was as follows: attention *α* = .90, clarity *α* = .90, and repair *α* = .86.

To demonstrated efficiency, we used the reduced version of TMMS - 24 (Salovey et al., 1995) with 8 items per dimension and with the objective of evaluating the self-perceived EI for each dimension. Students were asked to evaluate the degree to which they agree with each of the items on a Likert-type scale of 5 points (1 = No agreement, 5 = Strongly agree). This instrument classifies the dimensions attention, clarity, and repair in the values: low should improve (L), suitable, average (S), and high (H). The interpretation of the results are based on Table 1 (adaptation Fernandez-Berrocal et al., 2004).

### Procedure

Prior to the application of the questionnaire, a translation and adaptation to the Brazilian context were carried out with the participation of the NUPIC study group. The questionnaire was applied in an online format, using the Google Forms platform, and with the collaboration of professors. In each of the instances, they had received training on the questionnaire for its correct application.

This research was carried out during the months of September, October, November and December 2017 in the network of SESI-SP schools. Meetings were held with the director of the SESI-SP education division as well as with different advisors and teachers, along with the director of the study group (NUPIC) to elucidate the objectives of this research and ensure the collaboration of teachers and verify relevant permits were held by each educational unit.

## Results

### Self-perception of EI according to the gender of the participants

The study of descriptions in Table 3 shows a greater proportion of low scores in the three dimensions of the study in the case of women, with men only holding a slightly higher percentage of high scores in the dimension of emotional attention (17.7%).

**Table 3:** Gender proportions in each dimension of Emotional Intelligence Perceived (EIP); Test Chi-Squared and Effect sizes for Cramer’s V

		Men		Women		*Pearson's chi-square tests		Effect Size
		f	%	f	%			
Attention	Low	1630	29.2	2127	37.3	χ²	189.36	0.13
	Suitable	2963	53.1	3019	53.0	df	2	
	High	991	17.7	553	9.7	Sig.	0	
Clarity	Low	1657	29.7	2560	44.9	χ²	287.25	0.16
	Suitable	3391	60.7	2639	46.3	df	2	
	High	536	9.6	500	8.8	Sig.	0	
Repair	Low	1517	27.2	2468	43.3	χ²	322.86	0.16
	Suitable	3275	58.6	2567	45.0	df	2	
	High	792	14.2	664	11.7	Sig.	0	

*. The chi-square statistic is significant at the level. 05

The data obtained in this study regarding the dimensions of emotional self-perception regarding the gender of the participants show that for the female gender they have a higher proportion of low values than men, although the proportion of women, 53.0%, coincides with that of men, 53.1%. Regarding the clarity dimension, the proportion of women with *low* clarity, 44.9%, is much higher than that of men, 29.7%. These are unlike the case of suitable values where men represent 60.7%, and women 46.3%. The same applies to the repair dimension, but with a less pronounced difference. According to each of the dimensions of PEI studied, the proportions that suggest the need for improvement in the female gender are the dimensions of emotional regulation and emotional clarity.

Pearson’s chi-square test for categorical variables indicates that there is significance at the 0.05 level in the difference of proportions in the three dimensions. Therefore, there are significant differences between the population of men and women in terms of the PEI dimensions, and based on the size of the effect according to the Cramer’s V estimator, is considered small (Cohen, 1988) for the three dimensions.

### Self-perception of EI according to the level of studies of the participants

According to the data in Table 4 it is observed that there is a considerable proportion of participants who obtain low scores in all three dimensions and at all educational levels (between 30% and 40%). These results indicate a possible need for improvement in these capacities. The highest percentage for *low* values (39%) is given in the clarity dimension when students exceed 14 years of age, which corresponds to the 9th grade.

**Table 4:** Self-perception of emotional intelligence according to the level of studies of the participants. Test chi-squared and Effect sizes for Cramer’s V

		Level of studies of the participants								
		7th fundamental year		9th fundamental year		3rd year middle school		*Pearson's chi-square tests		Effect Size
		f	%	f	%	f	%			
Attention	Low	826	35.5	1523	35.6	1408	30.1	χ²	105.14	
	Suitable	1253	53.8	2271	53.1	2458	52.5	df	4	
	High	249	10.7	482	11.3	813	17.4	Sig.	.000^*^	0.07
Clarity	Low	780	33.5	1666	39.0	1771	37.8	χ²	21.09	
	Suitable	1311	56.3	2241	52.4	2478	53.0	df	4	
	High	237	10.2	369	8.6	430	9.2	Sig.	.000^*^	0.03
Repair	Low	751	32.3	1547	36.2	1687	36.1	χ²	28.85	
	Suitable	1218	52.3	2167	50.7	2457	52.5	df	4	
	High	359	15.4	562	13.1	535	11.4	Sig.	.000^*^	0.03

*. The chi-square statistic is significant at the level. 05

The *high* values in the attention dimension increase as they move through the different stages of the educational system (7th grade 10.7%, 9th grade 11.3%, and 3rd grade 17.4%). Considering that both variables are categorical, for the study of the dependency relationship between variables, chi-square statistics are used, which shows a relationship between the academic level and the three dimensions of PEI, although with a low effect size. Cohen (1988) values correspond to emotional Attention (*χ*^2^ = 105.144, *p* = 0.000, size effect = 0.07), emotional Clarity ((*χ*^2^ = 21.091, *p* = 0.000 size effect = 0.03), and emotional Repair ((*χ*^2^ = 28.851 = 0.000 size effect = 0.03). Therefore, there is a relationship between the dimensions of emotional intelligence and the age of the participants.

### Self-perception of EI and study of the relationship with parents’ occupations

The employment status of the parents (whose descriptive results were offered in Table 2) evidence of high employee status among fathers and mothers being slightly higher in the case of fathers 88.4%, than in the case of mothers 72.4%.

If we focus on the results provided by the mothers of the participants once the chi-square test has been carried out, it is observed that there is no relationship between the PEI dimensions and the employment situation, as shown in Table 5. A low proportion of the students surveyed obtain scores *high* in the dimensions of emotional clarity and emotional repair, with slightly higher results in these two dimensions when the mothers are actively employed. About the self-perception of emotional intelligence and the work status of parents, the study of the relationship between variables indicates that there is no significant relationship and the size of the effect is considered low (Cohen 1988), for the emotional attention dimension size effect = 0.01, clarity size effect = 0.03 and emotional repair size effect = 0.03.

**Table 5:** Self-perception of emotional intelligence according to the employment situation of mothers and fathers. Test chi-squared and Effect sizes for Cramer’s V

		Situation mother								
		WORK		**DK/NR		NO WORK		*Pearson's chi-square tests		Effect Size
		f	%	f	%	f	%			
Attention	Low	2720	33.0	17	37.0	1020	33.9	χ²	6.08	
	Suitable	4362	53.0	19	41.3	1601	53.2	Df	4	
	High	1148	13.9	10	21.7	386	12.8	Sig.	0.19	0.02
Clarity	Low	3063	37.2	20	43.5	1134	37.7	χ²	2.30	
	Suitable	4412	53.6	20	43.5	1598	53.1	Df	4	
	High	755	9.2	6	13.0	275	9.1	Sig.	0.68	0.01
Repair	Low	2952	35.9	18	39.1	1015	33.8	χ²	6.03	
	Suitable	4207	51.1	23	50.0	1612	53.6	Df	4	
	High	1071	13.0	5	10.9	380	12.6	Sig.	0.19	0.02
		Situation father								
		WORK		**DK/NR		NO WORK		*Pearson's chi-square tests		Effect Size
		f	%	f	%	f	%			
Attention	Low	3362	33.4	28	39.4	367	31.7	χ²	5.04	
	Suitable	5319	52.9	30	42.3	633	54.7	df	4	
	High	1373	13.7	13	18.3	158	13.6	Sig.	.28^a^	0.01
Clarity	Low	3738	37.2	27	38.0	452	39.0	χ²	2.47	
	Suitable	5385	53.6	36	50.7	609	52.6	df	4	
	High	931	9.3	8	11.3	97	8.4	Sig.	.65^a^	0.01
Repair	Low	3520	35.0	29	40.8	436	37.7	χ²	5.50	
	Suitable	5218	51.9	34	47.9	590	50.9	df	4	
	High	1316	13.1	8	11.3	132	11.4	Sig.	.24^a^	0.01

*. The chi-square statistic is significant at the level. 05

**.(DK) Don’t Know (NR) No Reply

After carrying out a statistical analysis considering these groups: both parents work; only one of them works or neither works with PEI variable (Table 6); we observed that the group of students that both parents work showed the most percentage approx. 65.04% between the groups surveyed.

**Table 6:** Self-perception of emotional intelligence according to each of these groups: both parents work, only one of them works or neither works. Pearson's chi-square test and Effect sizes for Cramer’s V

		Both parents work		Father work mother no		Mother work father no		Neither works		**DK/NR		*Chi-square		EffectSize
		f	%	f	%	f	%	f	%	f	%			
f	11283	7339	65.04	2674	23.70	834	7.39	321	2.84	115	1.02			
Attention	Low	2441	33.3	905	33.8	257	30.8	109	34.0	45	39.1	χ²	16.59	0.03
	Suitable	3868	52.7	1435	53.7	471	56.5	160	49.8	48	41.7	df	8	
	High	1030	14.0	334	12.5	106	12.7	52	16.2	22	19.1	Sig.	.035^*^	
Clarity	Low	2707	36.9	1015	38.0	333	39.9	116	36.1	46	40.0	χ²	7.35	0.01
	Suitable	3946	53.8	1419	53.1	438	52.5	171	53.3	56	48.7	df	8	
	High	686	9.3	240	9.0	63	7.6	34	10.6	13	11.3	Sig.	.49	
Repair	Low	2617	35.7	886	33.1	311	37.3	124	38.6	47	40.9	χ²	13.62	0.02
	Suitable	3753	51.1	1445	54.0	425	51.0	163	50.8	56	48.7	df	8	
	High	969	13.2	343	12.8	98	11.8	34	10.6	12	10.4	Sig.	.09	

*. The chi-square statistic is significant at the level. 05.

**.(DK) Don’t Know (NR) No Reply

In the attention dimension, the group that reaches the highest percentage is denominated *suitable* and corresponds to the students whose mother worked, but the father did not work, 56.5%. For the students who responded that their father worked, but their mother did not, the data shows the highest scores in the repair dimension, where the proportion of students who got the adequate repair of *suitable* was 54.0%. However, the data to be highlighted were recorded when both parents were actively working with repair levels of *high* at 13.2%.

In the group where both parents work, they present similar percentages in the three dimensions in adequate assessment of *suitable*, between 51 and 54%. Regarding the values of *low*, a higher percentage in the clarity dimension stands out with 36.9%. In the group where the father works and the mother does not work, with 23% of the total analyzed, there are similar percentages in the three dimensions in the appropriate assessment of *suitable,* which coincides with the group previously analyzed. It should be noted that the highest proportion of the values of *low* are in the clarity dimension at 38.0%. In the group where the mother works and the father does not work, which represents 7.39 %, the good results that stand out in the attention dimension are with *suitable,* 56.5%. In the clarity dimension showed low values with 39.9% of the total of groups evaluated, the same occurs in the repair dimension with one 37.3%. On the other hand, we look that there a relation between the groups where none of the parents work and the attention dimension. The group of students who do not know, or do not answer because the reason for this response is unknown, is not analyzed.

## Discussion and conclusions

For years, there has been a concern for the development of emotional competencies and reflections on the importance of promoting the development of emotional and social competences in the educational and social environment (Fundaci, & Clouder, 2008). The new models of positive development advocate the promotion of healthy adolescent development, and that the school will be an ideal context to promote emotional competencies (Pertegal et al., 2010).

Conducting research on the relationships between PEI and factors such as gender has implications for an individual’s description of their emotional development, while also informing us about the detailed needs for intervention within the classroom. In the present study, differences are perceived between males and females with respect to their emotional capacities being consistent with previous research (Gartzia et al., 2012). In addition, some research shows that certain areas of the brain dedicated to emotional processing may be larger in women than in men and that these differences are affirmed in the differentiation in the processing of emotions (Baron-Cohen, 2003; Gur et al., 2002).

In this study, women present low values in the three PEI dimensions. The values suggest that female subjects need for improvement lies in the dimensions of emotional regulation (43.3%), and of emotional clarity (44.9%). Similar survey results show high levels of reparation in terms of problem-solving and high social and emotional competence in men over women (Jones et al., 2002).

The female group of this study has a high percentage in the attention dimension in its value *high*, which is characteristic of people who care intensely about their emotions. Based on this evidence, it could highlight the great urgency with which women need to develop verbal skills and to be more competent in articulating feelings, which would allow them to have more resources and information about the emotional world (Brody, 1993). This data is in line with previous studies in which in the sample of adolescents who reported higher levels of attention in the case of women than in male students (Salguero et al., 2012). In contrast, some authors such as (Guastello & Guastello, 2003) or (Schutte et al., 1998) argue that gender differences are getting smaller, due to socio-cultural work in terms of gender roles and stereotypes.

In other studies, the EI self-report tests show that women show higher scores in emotional attention and empathy, while men score higher in emotional regulation (Austin et al., 2005; Brackett et al., 2004; Schutte et al., 1998). These results do not correspond entirely to the sample participating in this study. The sociocultural context of the sample may be the origin of this difference in the sample.

According to the results related to the level of studies of the participants, the sample indicates that there is a considerable proportion of participants who obtain low scores in all three dimensions, results that indicate a possible need for improvement in these capacities. We consider a prominent fact that the values considered *high* in the attention dimension increase as they move through the different stages of the education system. Data that does not fit other studies that show younger students’ express emotions such as sadness or anger with higher frequency than older students (Zeman and Garber, 1996; Zeman & Shipman, 1996).

Therefore, an aspect important to be considered at the high school is the need to take better care of the emotional attention the students since that obtaining high values in emotional attention doesn't mean that sustains the emotional balance in the adolescents. Various studies have shown that an excessive level of emotional attention without understanding its causes or consequences, can become harmful, generating emotional fatigue (Extremera, and Fernández-Berrocal, and Durán, A., 2003).

Regarding the aspects of the study related to the relationship between parental employment and high levels of reparation, especially in the case of girls, previous studies have shown that parents tend to talk more about emotions with their daughters than with sons (Trinidad & Johnson, 2002).

Research carried out in the Brazilian context, such as those by Andrade Neta et al., 2017, puts into perspective the need, within the education sector, to evolve both in educational policies and in the strategic vision of the high schools and the social education competencies and emotional of the students. Which is an internationally recognized need (Gurgel et al., 2016). Considering the development of EI at work or at school offers training and response to social problems, but interventions should be based solely on respectable theories and careful definition and analysis of previous emotional competencies (Zeidner et al., 2009).

In this study, based on the students’ self-perception, we have studied the sociodemographic factors that should undoubtedly be considered when proposing quality socio-educational interventions, since the data shows that beliefs about success in emotional regulation are positively associated with results. Thus, the emotional factor can be considered a crucial point in improving education in schools (Bigman et al., 2016).

However, in future research, it would be interesting to use other instruments, previously adapted and translated for the Brazilian context, as has been done in the present study with the TMMS-24 instrument together with the NUPIC research team of the University of Sao Paulo. Instruments such as the MSCEITT (Mayer-Salovey-Caruso Emotional Intelligence Test) or the TIEFBA (Emotional Intelligence Test of the Botin Foundation for Adolescents) that mitigates some of the difficulties that arise when measuring self-report and adapt to the ages and context of the sample of population studied.

Therefore, in the application of training programs in EI we have to take into account a whole series of socio-cultural aspects, based on a series of initial measures that allow not only to observe the perceptions of the participants but also to influence the willingness of participants and beneficiaries to participate in this type of intervention, being protected by a solid theoretical basis and under a rigorous study that allows to verify its effectiveness.

Consequently, the present study has implied the promotion of future research to demonstrate the impact of EI on well-being, the improvement of academic performance and the development of the ability to coexist (Fincias et al., 2018; Fundaci and Clouder, 2008).

## Data Availability

All data generated and analyzed during this study will be treated with total confidentiality. The dataset supporting the conclusions of this article is available by request to the authors.

## Abbreviations

EI: Emotional Intelligence
MSCEITT: Mayer-Salovey-Caruso Emotional Intelligence Test
NUPIC: Núcleo de Pesquisas em Inovações Curriculares, Faculdade de Educação da Universidade de São Paulo
PEI: Perceived emotional intelligence
SARESP: School Performance Evaluation System of the State of São Paulo
SESI-SP: Serviço Social da Indústria- of São Paulo, Brasil
TIEFBA: Emotional Intelligence Test Botin Foundation for Adolescent
